# A 13-lipoxygenase, TomloxC, is essential for synthesis of C5 flavour volatiles in tomato

**DOI:** 10.1093/jxb/ert382

**Published:** 2014-01-22

**Authors:** Jiyuan Shen, Denise Tieman, Jeffrey B. Jones, Mark G. Taylor, Eric Schmelz, Alisa Huffaker, Dawn Bies, Kunsong Chen, Harry J. Klee

**Affiliations:** ^1^Horticultural Sciences, University of Florida, PO Box 110690, Gainesville, FL 32611-0690, USA; ^2^Laboratory of Fruit Quality Biology/The State dgriculture Ministry Laboratory of Horticultural Plant Growth, Development and Quality Improvement, Zhejiang University, Zijingang Campus, Hangzhou 310058, PR China; ^3^Department of Plant Pathology, University of Florida, Gainesville, FL 32611-068, USA; ^4^United States Department of Agriculture-Agricultural Research Service, Center for Medical Agricultural and Veterinary Entomology, 1700 SW 23rd Drive, Gainesville, FL 32608, USA

**Keywords:** C5 volatiles, hydroperoxide lyase, jasmonic acid, 13-lipoxygenase, tomato, wounding, *Xanthomonas campestris* pv. *vesicatoria*.

## Abstract

C5 volatile compounds, derived from fatty acids, are among the most important contributors to consumer liking of fresh tomatoes. Despite their important roles in flavour, the genes responsible for C5 volatile synthesis have yet to be identified. This work shows that their synthesis is catalysed in part by a 13-lipoxygenase (LOX), TomloxC, the same enzyme responsible for synthesis of C6 volatiles. C5 synthesis is independent of hydroperoxide lyase (HPL); moreover, HPL knockdown significantly increased C5 volatile synthesis. This LOX-dependent, HPL-independent pathway functions in both fruits and leaves. Synthesis of C5 volatiles increases in leaves following mechanical wounding but does not increase in response to infection with *Xanthomonas campestris* pv. *vesicatoria*. Large reductions in C5 and C6 volatiles in antisense *TomloxC* knockdown plants were observed but those reductions did not alter the development of disease symptoms, indicating that these volatiles do not have an important defensive function against this bacterial pathogen.

## Introduction

Plants are capable of synthesizing and releasing an array of volatile organic compounds derived from a diverse set of primary metabolites that include amino acids, fatty acids, and terpenes (Schwab *et al.*, 2008; Klee, 2010). These volatiles have countless functions in intra- and interkingdom interactions, including those with humans (Tieman *et al.*, 2012), animals, insects (Dicke and Baldwin, 2010), and microorganisms (Christensen and Kolomiets, 2011).

In the context of human preference, consumption, and health, food-derived volatiles are of particular interest. Flavour improvement requires both knowledge of the biosynthetic pathways for flavour volatiles and knowledge of the genes that regulate synthesis. Some of the most important volatiles contributing to consumer liking of tomatoes are the C5 volatiles such as 1-penten-3-one, (*E*)*-*2-pentenal, 3-pentanone, 1-pentanol, and 1-penten-3-ol (Tieman *et al.*, 2012). Despite their importance to tomato flavour, the pathway and enzyme(s) responsible for their synthesis from fatty acids has not been determined. However, researche performed in other plant species strongly suggests involvement of one or more lipoxygenases (Salch *et al.*, 1995). The lipoxygenase pathway is well known for generating C6 volatiles that are abundant in tomato fruits, most notably (*Z*)-3-hexenal, hexenol, hexanal, and hexanol (Chen *et al.*, 2004).

The peroxidation of C18 polyunsaturated fatty acids, such as linolenic and linoleic acids, is initially catalysed by lipoxygenases (LOXs). LOX enzymes can act upon polyunsaturated fatty acids at either the C9 or C13 position, yielding two groups of hydroperoxides; the enzymes are referred to as 9- and 13-LOXs respectively, depending on which position is oxygenated. 9-LOXs comprise a subfamily of proteins that share high amino-acid sequence identity (~60%) to one another, but 13-LOXs are more diverse, sharing only ~35% sequence identity among themselves (Andreou and Feussner, 2009; Park *et al.*, 2010). These two types of LOX enzymes can largely be distinguished by the expression patterns of the genes encoding them, as well as their substrate preferences and subcellular localizations. While this categorization is typically clear, LOX enzymes with dual C9 and C13 positional specificity have also been described (Kim *et al.*, 2003).

**Fig. 1.**
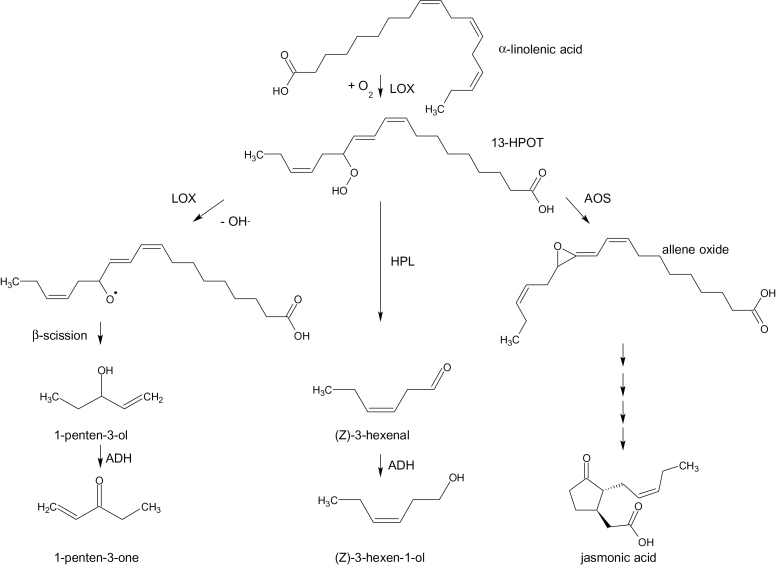
Pathways for synthesis of linolenic acid products in tomato. The pathway for synthesis of C5 volatiles is based on the proposed soybean pathway (Salch *et al.*, 1995). ADH, alcohol dehydrogenase; AOC, allene oxide cyclase; AOS, allene oxide synthase; HPL, hydroperoxide lyase; LOX, lipoxygenase; 13-HPOT, 13(*S*)-hydroperoxy-9(Z),11(E),15(*Z*)-octadecatrienoic acid.

In the case of linolenic acid (18:3) metabolism, LOX generates 13(*S*)-hydroperoxy-9(*Z*),11(*E*),15(*Z*)-octadecatrienoic acid (13-HPOT). Three potential fates for hydroperoxides such as 13-HPOT have been described in the literature (Fig. 1). For 13-HPOT in particular, the first step in the jasmonic acid (JA) pathway is catalysed by allene oxide synthase. This pathway includes a number of biologically active oxylipins, including jasmonic acid and is a critical component of responses to pathogens, wounding and herbivory (Schaller and Stintzi, 2009). Entry into a second hydroperoxide pathway is catalysed by hydroperoxide lyase (HPL) (Porta and Rocha-Sosa, 2002; Schwab *et al.*, 2008). In the HPL branch, the hydroperoxides are oxidatively cleaved into C6 aldehydes, including hexanal, (*E*)-2-hexenal, and (*Z*)-3-hexenal. These aldehydes are subsequently reduced to their corresponding alcohols by alcohol dehydrogenase (Speirs *et al.*, 1998). Major C6 alcohol products can also be released as modified volatiles, such as (*Z*)-3-hexen-1-yl acetate, following an acylation by BAHD acyltransferases (D’Auria *et al.*, 2007).

Compared to the well-known C6 volatile biosynthesis pathway, the synthesis of C5 compounds is less established. For example, *in vitro* enzymic formation of 1-penten-3-ol (pentenol) and 1-penten-3-one (pentenone) from soybean (*Glycine max*) is dependent on 13-HPOT and blocked by LOX inhibitors (Salch *et al.*, 1995). A pathway was proposed in which LOX oxygenates 13-HPOT, followed by a β-elimination, which generates the C5 compounds. C5 compounds such as 1-penten-3-ol and (*Z*)-2-pentenal were also observed to accumulate at higher levels in HPL-depleted transgenic potato and *Arabidopsis* plants (Vancanneyt *et al.*, 2001; Salas *et al.*, 2006), but the mechanism was not identified.

Metabolites in the LOX pathway are considered to mediate communication both within and between biological kingdoms. Beyond their roles in flavour (Goff and Klee, 2006), mounting evidence indicates that oxylipins, including C6 green leafy volatiles (GLVs) and jasmonates, also play important roles in plant responses to biotic stress (Weber, 2002; Farmer *et al.*, 2003; Kachroo and Kachroo, 2009). For example, aerial treatment of *Arabidopsis thaliana* seedlings with low concentration of (*E*)-2-hexenal induced expression of several genes associated with defence responses (Bate and Rothstein, 1998). (*E*)-2-hexenal and (*Z*)-3-hexenol, which are released from lima bean leaves inoculated with pathogenic bacteria, strongly inhibit *Pseudomonas syringae* growth (Croft *et al.*, 1993). Likewise, pretreatment with C6-aldehydes retarded disease symptom development in *Arabidopsis* inoculated with *Botrytis cinerea* (Kishimoto *et al.*, 2005). Interestingly, the emission of GLVs induced by herbivore attack can lead to higher mortality of the herbivores via attracting the natural enemies of herbivores (Shiojiri *et al.*, 2006). There are also many reports supporting the role of JA and its precursor 12-oxo-phytodienoic acid in signal transduction of defence responses (Stintzi *et al.*, 2001; Kachroo and Kachroo, 2009; Park *et al.*, 2013).

Even though 13-LOX enzyme catalysis is required for jasmonate and C6 volatile formation, it was found that distinct 13-LOXs specialized in the generation of C6 GLVs and jasmonates, respectively. For instance, in potato the production of GLVs depended on LOX-H1 enzyme activity while JA biosynthesis was not reduced in LOX-H1 depleted transgenic plants (Leon *et al.*, 2002). A similar study in *Nicotiana attenuata* demonstrated that silencing of *NaLOX3*, which is homologous to potato *LOX-H3*, suppressed JA synthesis but not the release of GLVs (Halitschke and Baldwin, 2003), while reduced *NaLOX2* expression led to strongly decreased GLVs production without effects on JA-related metabolites (Allmann *et al.*, 2010). In tomato (*Solanum lycopersicum*), there are six LOX-encoding genes, *TomloxA–F* (Chen *et al.*, 2004; http://solgenomics.net/; Supplementary Fig. S1, available at *JXB* online). *TomloxA*, *TomloxB*, and *TomloxE* are predicted to encode 9-LOX enzymes and are not involved in C6 volatile synthesis (Griffiths *et al.*, 1999; Chen *et al.*, 2004). *TomloxC* and *TomloxD* are predicted to be chloroplast localized and encode 13-LOX enzymes (Chen *et al.*, 2004). *TomloxC* is expressed most highly in ripening fruits while *TomloxD* is expressed in fruits and leaves. *TomloxC* encodes the LOX enzyme that is essential for generation of fruit C6 volatiles (Chen *et al.*, 2004). Expression of another 13-LOX, TomloxF, sharing 76% amino acid identity with TomloxC, is stimulated by infection with *Pseudomonas putida* BTP1 to produce 13-HPOT and 13-hydroxy-octadecatrienoic acid (13-HOT) (Mariutto *et al.*, 2011). The activities of TomloxC and TomloxF in the generation of JA and GLVs remain to be characterized.

This work presents evidence for a critical role for TomloxC in tomato fruit and leaf biosynthesis of C5 compounds, such as 1-penten-3-ol, 1-penten-3-one, pentanal, (*Z*)-2-penten-1-ol, and 1-pentanol. While C5 and C6 GLVs are induced by wounding in a TomloxC-dependent reaction, they are not induced by infection by the bacterial pathogen, *Xanthomonas campestris* pv*. vesicatoria* (*Xcv*) 93-1.

## Materials and methods

### Plant materials

The full length open reading frame of *TomloxC* was cloned into a vector containing the constitutive Figwort mosaic virus 35S promoter in the antisense orientation; the transformation vector was introduced into the wild-type tomato control (*S. lycopersicum* cv. M82) as previously described (Tieman *et al.*, 2012). An RNAi construct for LeHPL was introduced into *S. lycopersicum* cv. Flora-Dade., A 330-bp fragment comprising bases 562–881 of the open reading frame in the sense orientation and a 595-bp fragment comprising bases 562–1154 of the open reading frame in the antisense orientation were expressed under the control of the Figwort mosaic virus 35S promoter.

The transgenic and control plants were grown in the field (University of Florida North Florida Research and Education Center, Suwannee Valley) and greenhouse (University of Florida campus in Gainesville, FL) in spring and fall seasons to obtain the fruits for the analysis of volatile compounds. The tomato plants for pathogen experiments were grown in small pots in the greenhouse under ambient light with daily hand watering. Four-week-old plants with five fully expanded leaves were subjected to pathogen inoculation; the second and third true leaves were harvested for subsequent experiments. All experiments were repeated at least three times, and typical results are shown.

### Inoculation

*X. campestris* pv. *vesicatoria* Xv 93-1 (Astua-Monge *et al.*, 2000) was grown overnight in nutrient broth (0.8% BBL, Becton Dickinson, Cockeysville, MD) at 30° C and diluted with sterilized tap water and the inoculum was adjusted to 1×10^6^ colony-forming units ml^–1^ and Silwet 77 was added to a final concentration of 0.025%. For M82 and LoxC-AS plants, the inoculation was performed by dipping the plants individually in suspension for 15 s, while mock-inoculated plants were dipped in the same solution without bacteria. All plants were maintained in the greenhouse throughout the duration of the experiment.

### Disease assays

Electrolyte leakage was measured as described previously (Lund *et al.*, 1998) on the three most apical leaflets of the second and third true leaves from three plants per genotype per day. Bacterial growth was determined as described by (Lund *et al.*, 1998) with adjustment. Leaf discs (2cm^2^) were excised from the three most apical leaflets of the second and third true leaves from three plants per genotype per day, and ground in 2ml of sterile tap water. Serial dilutions of the bacterial solution were plated on nutrient agar plates. After 2 d at 28° C, colony-forming units were counted for each time point.

### Jasmonic acid and free fatty acid quantification

JA and free fatty acid levels in infected and mock-infected leaves were methylated, partially purified by vapour phase extraction, and quantified by gas chromatography/isobutane chemical-ionization mass-spectrometry as previously described (Schmelz *et al.*, 2004). A subset of the leaves as described above for the electrolyte leakage assays were used. Each data point is the average of three biological replicates.

### Volatile analysis

The three apical leaflets of the second and third true leaves from three plants inoculated with pathogen per genotype per day were used for volatile collection. Wounded leaves were obtained by chopping with a sharp knife and crushing, followed by volatile collection. Volatiles were collected from leaves and fruits as previously described (Tieman *et al.*, 2006). Briefly, headspace volatiles were collected from chopped fruits or wounded or unwounded leaves for 1h and analysed by gas chromatography. Volatiles were identified by retention time and mass spectrometry compared to known standards.

### RNA isolation and quantification

Total RNA from leaf tissue collected 30min after wounding and ripe fruit was extracted using a RNeasy Plant Mini Kit and treated with DNase (Qiagen) to remove genomic DNA. Transcripts of *TomloxC* (Solyc01g006540), *TomloxD* (Solyc03g122340), and *TomloxF* (Solyc01g006560) from 200ng of total RNA were measured using a Power SYBR Green RNA-to-C_T_ 1-Step Kit (Life Technologies). The primers for real-time reverse-transcription PCR were: TomloxC: forward 5′- GCAATGCATCATGTGTGCTA-3′ and reverse 5′- GTAAATGTCGAATTCCCTTCG-3′; TomloxD: forward 5′- GGCTTGCTTTACTCCTGGTC -3′ and reverse 5′- AAATCAAAGCGCCAGTTCTT-3′; and TomloxF: forward 5′- CCGAATCAAAGGGTGACTTT -3′ and reverse 5′- GGTCTGTGATGATCGATTGC-3′.

For measuring the mRNA level of *LeHPL* (Solyc07g049690) in tomato fruits, a Taqman probe strategy was applied to the real-time PCR using One-step RT-PCR reagents (Life Technologies). The primers and Taqman probes were designed using the Genscript website (http://www.genscript.com/). The following primers and probe were used: LeHPL: forward 5′-AGCTACGGATTGCCGTTAGT-3′ and reverse 5′-TTTCCATTCTCTTGGTGAAGAA-3′; and LeHPL Taqman probe: (5′-6-FAM)–ATCGATCCGCGATTGGC CC-(3′-TAMRA).

The reactions of quantitative PCR were performed using a StepOnePlus Real-time PCR system and the data were subjected to absolute quantification using a standard curve derived from plasmid DNA ranging from 10^3^ to 10^8^ copies per sample.

### Statistical analysis

Unpaired Student’s t-test was applied to the two-sample comparisons. For multiple comparisons one-way ANOVA was used. If the ANOVA was significant (*P* ≤ 0.05), the Newman–Keuls test was performed to detect significant differences between groups.

## Results

### The pathway for synthesis of C5 and C6 volatiles in tomato fruits

**Fig. 2.**
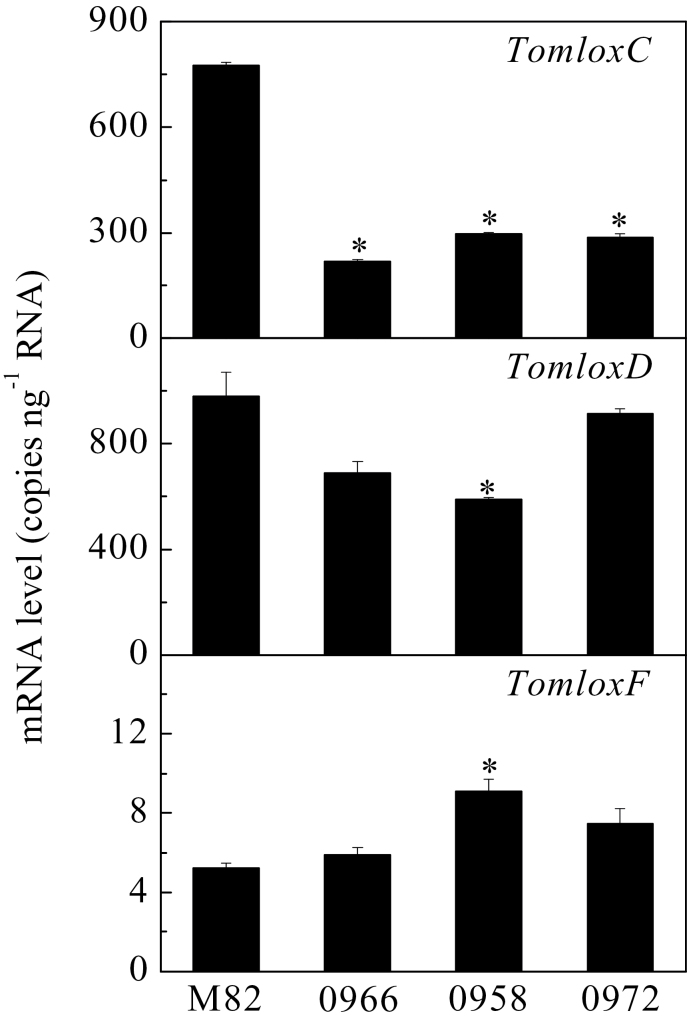
Transcript levels of *TomloxC*, *TomloxD*, and *TomloxF* in ripe fruits. RNA was extracted from fruits of wild-type (M82) and three independent transgenic lines containing the *LoxC-*AS construct. Transcripts were quantified by quantitative reverse-transcription PCR. Values are mean ± standard error of three replicates. Significant differences are indicated with asterisks above the bars: **P ≤ 0.01.

While previous work has established an unambiguous role for *TomloxC* in synthesis of C6 volatiles in tomato fruits (Chen *et al.*, 2004; Tieman *et al.*, 2012), the pathway(s) for synthesis of C5 volatiles is less clear. Given that C5 volatiles are highly correlated with consumer preferences for tomato fruits (Tieman *et al.*, 2012), the first step towards flavour improvement is to understand the pathway and the gene(s) responsible for their synthesis. It has been hypothesized that LOX enzymes catalyse chain cleavage of hydroperoxides to release pentenols (Salch *et al.*, 1995; Gardner *et al.*, 1996) (Fig. 1). This reaction contrasts with that for synthesis of C6 volatiles that are generated by HPL-mediated cleavage of the same hydroperoxides. There are three putative 13-lipoxygenase-encoding genes in tomato: *TomloxC*, *TomloxD*, and *TomloxF* (Supplementary Fig. S1). This work used quantitative reverse-transcription PCR to determine the relative levels of expression of these three genes in ripe tomato fruits from three independent transgenic events (Fig. 2).

The results indicated that, in ripe fruits in an M82 background, *TomloxD* and *TomloxC* are the most highly expressed 13-LOX-encoding genes with *TomloxF* being expressed at a far lower level. *TomloxC* gene expression was blocked by introducing a full-length, constitutively expressed antisense cDNA into *S. lycopersicum* cv. M82. The cDNA is 78% identical to *TomloxF* and 31% identical to *TomloxD* within the region used in the antisense construct. Three independent transgenic lines (*LoxC*-AS) with greatly reduced *TomloxC* mRNA levels, lines 0958, 0966 and 0972 were chosen for further characterization. There was no reduction in *TomloxF* RNA observed in any of the three lines. There was a small but significant reduction in *TomloxD* RNA in line 0958. The effects on C6 volatile synthesis in Line 0966 have previously been described (Tieman *et al.*, 2012). The antisense construction specifically suppressed *TomloxC*, as no reduction in expression of the two closest homologues, *TomloxD* and *TomloxF* was observed in ripe fruits from this line (Fig. 2). Fruits from the three lines were screened for C5 and C6 volatile synthesis.

**Fig. 3.**
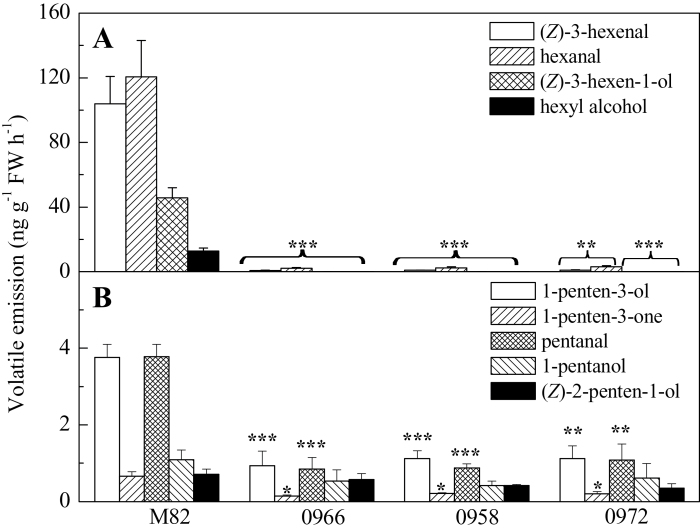
Reduced *TomloxC* expression in fruits decreases C5 and C6 volatile emissions in ripe fruits. (A) Concentrations of C6 compounds [hexanal, (*Z*)-3-hexenal, hexyl alcohol, and (*Z*)-3-hexen-1-ol] in wild-type (M82) and three *LoxC*-AS lines. (B) Concentrations of C5 volatiles [1-penten-3-ol, 1-penten-3-one, pentanal, 1-pentanol, and (*Z*)-2-penten-1-ol]. Values are mean ± standard error. Significant differences are indicated with asterisks above the bars: *P ≤ 0.05, **P ≤ 0.01, ***P ≤ 0.001.

Consistent with the results of Chen *et al.* (2004) and the current study group’s previous results (Tieman *et al.*, 2012), suppression of *TomloxC* expression resulted in significantly decreased emissions of all the measured C6 volatiles (hexanal, (*Z*)-3-hexenal, hexanol, and (*Z*)-3-hexen-1-ol) in each antisense line (Fig. 3A). The contents of multiple C5 volatiles including 1-penten-3-ol, 1-penten-3-one, and pentanal were also significantly reduced in *TomloxC* antisense lines; the emissions of (*Z*)-2-penten-1-ol and 1-pentanol were also decreased but not significantly (Fig. 3B). These results are consistent with a specific role for TomloxC in both fruit C5 and C6 volatile synthesis.

**Fig. 4.**
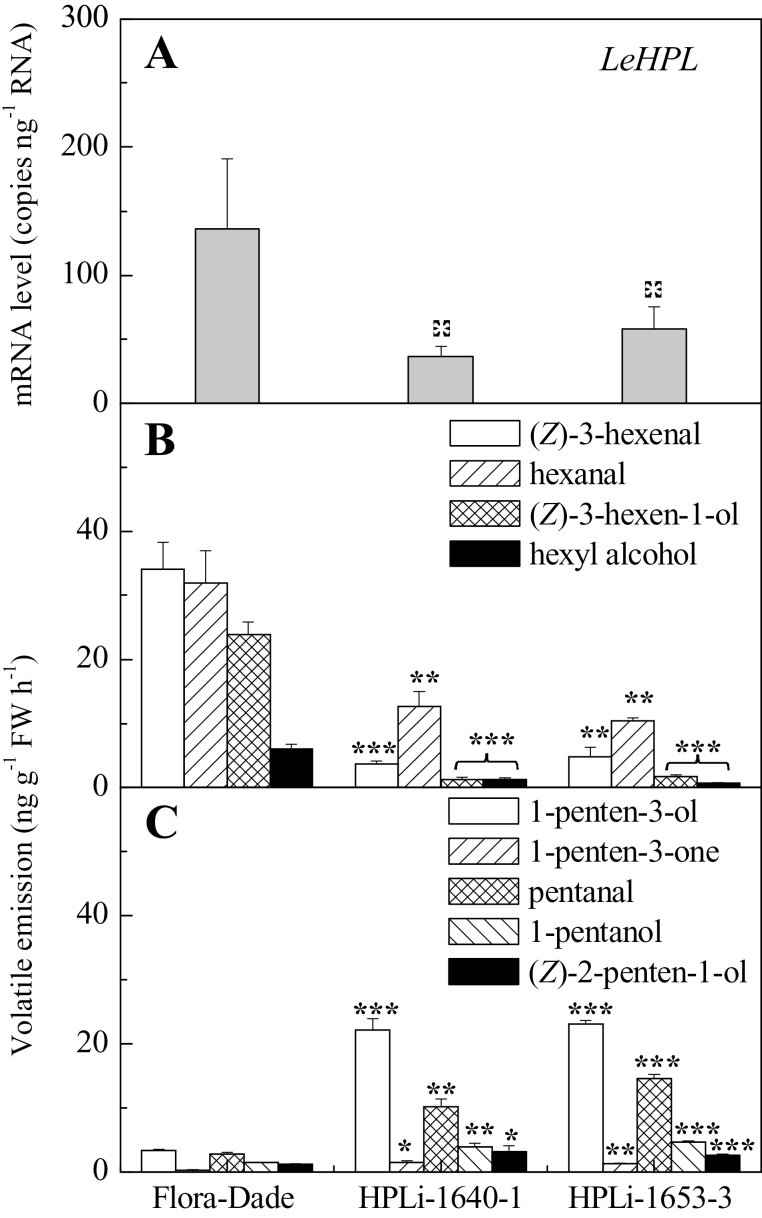
*LeHPL* downregulation decreases C6 volatiles and increases C5 fruit volatiles. (A) *LeHPL* transcript levels in fruits from wild type (Flora-Dade) and two transgenic lines. (B) C6 volatile emissions from ripe fruits. (C) C5 volatile emissions from ripe fruits. Values are mean ± standard error. Significant differences are indicated with asterisks above bars: *P ≤ 0.05, **P ≤ 0.01, and ***P ≤ 0.001.

If generation of C5 volatiles is independent of HPL, knockdown of *HPL* expression may lead to more C5 volatile synthesis. Such effects have been observed in an antisense potato knockdown line (Vancanneyt *et al.*, 2001) and in an *Arabidopsis* T-DNA knockout line (Salas *et al.*, 2006). To test this hypothesis, the current work produced transgenic plants expressing an RNAi transcript targeting *LeHPL*, the gene encoding an enzyme that cleaves 13-HPOT to generate C6 aldehydes (Howe *et al.*, 2000). Two independent transgenic events with significantly reduced *HPL* transcript were identified for further characterization (Fig. 4A). These lines both exhibited significantly reduced C6 volatile emissions in fruits relative to the non-transgenic control, Flora-Dade (Fig. 4B). In contrast, emissions of C5 volatiles were significantly increased relative to the control (Fig. 4C). The higher rate of C5 emissions is likely due to an increased pool of 13-hydroperoxide accumulation in fruits with reduced LeHPL activity. Thus, synthesis of these important flavour-associated volatiles is at least partially dependent upon TomloxC activity and is independent of LeHPL activity.

### The pathway for synthesis of C5 and C6 volatiles in tomato leaves

The production of GLVs, including C6 compounds, by leaves has been correlated with the response of the plant to external stresses including mechanical damage, herbivory, and pathogen attack (Howe and Jander, 2008; Dicke and Baldwin, 2010). In response to such stimuli, induced LOX enzymes metabolize fatty acids to JA and C6 volatiles (Halitschke *et al.*, 2004; Shiojiri *et al.*, 2006). In order to assess the importance of *TomloxC* in GLV synthesis, this work subjected leaves to two forms of stress, wounding and challenge with the tomato pathogen *Xcv*.

**Fig. 5.**
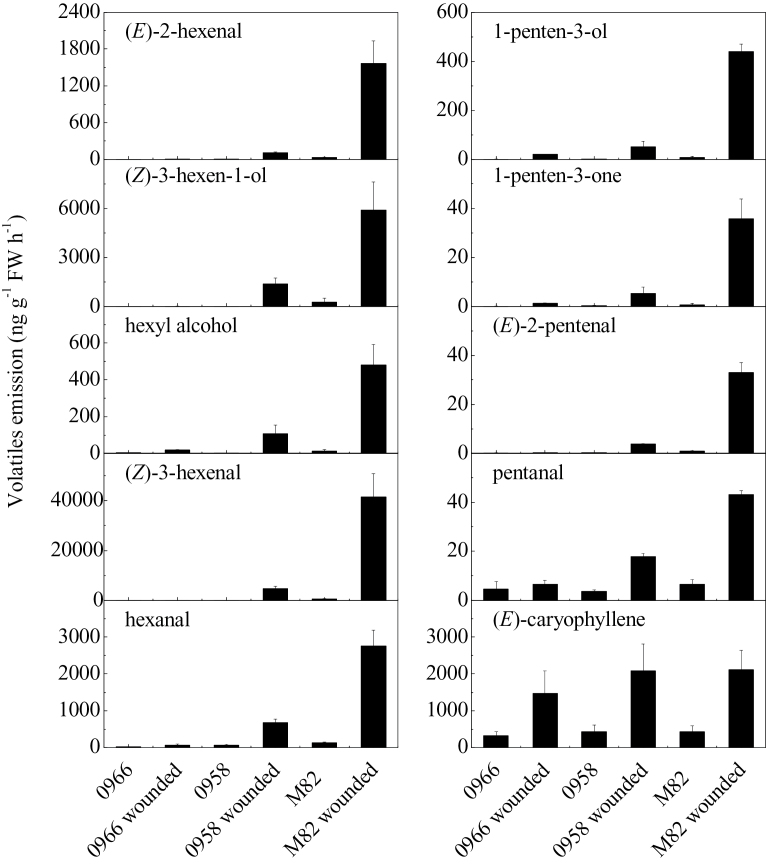
Release of C5 and C6 volatiles in M82 and *LoxC*-AS lines in response to wounding. Wounded leaves were obtained by chopping with a sharp knife and crushing, followed by a 1-h volatile collection, which were quantified. (*E*)-caryophyllene is also shown as an example of an unrelated terpenoid wound-induced volatile. Values are mean ± standard error.

**Fig. 6.**
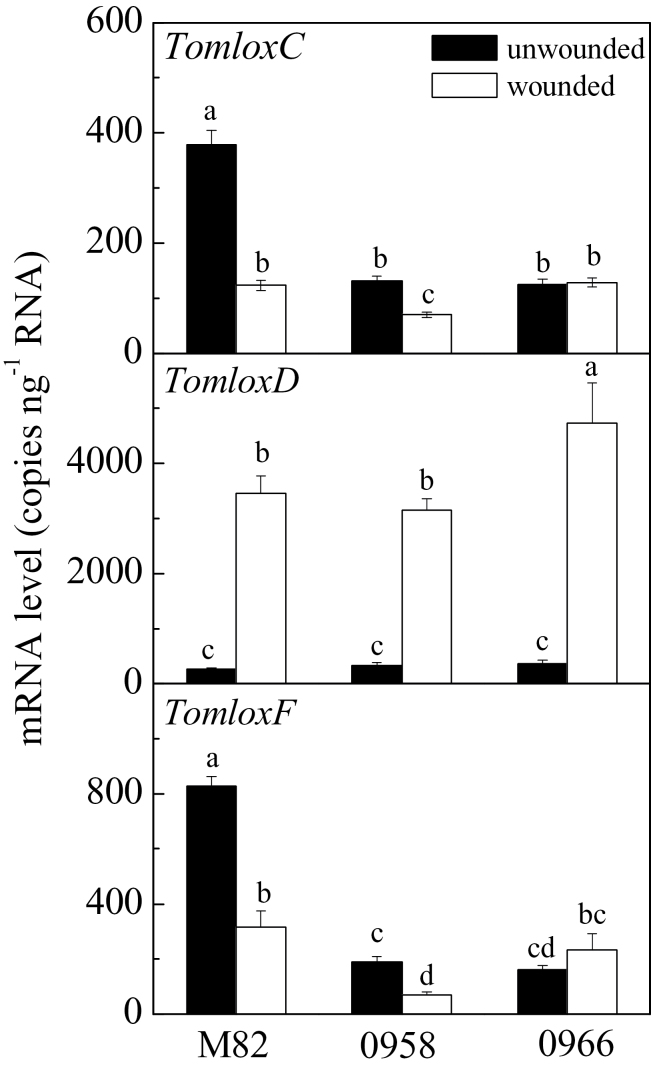
Transcript levels of *TomloxC*, *TomloxD*, and *TomloxF* in M82 and transgenic (*LoxC*-AS) wounded or unwounded leaves. Transcripts were quantified by quantitative reverse-transcription PCR. Values are mean ± standard error of two technical replicates of three biological replicates. Different letters above bars indicate significant differences (*P* ≤ 0.05, one-way ANOVA).

In the absence of stress, leaves of wild-type M82 plants synthesized very low levels of both C5 and C6 volatiles (Fig. 5). Upon wounding, there was a large increase in synthesis of all of the measured volatiles. In contrast to ripe fruits, *TomloxC*, *TomloxF*, and *TomloxD* RNAs were present at roughly equivalent levels in unwounded leaves (Fig. 6). Thirty minutes after wounding, *TomloxC* and *TomloxF* RNA contents were significantly reduced while that of *TomloxD* very significantly increased, making up approximately 90% of the 13-LOX RNA. In contrast to fruits, there were reductions in both *TomloxC* and *TomloxF*, but not *TomloxD* RNA levels in transgenic leaves (Fig. 6). The lack of effect of the transgene on fruit *TomloxF* RNA may be due to its extremely low expression in that tissue, the RNA being approximately 80-fold more abundant in leaves. Wound-induced GLV synthesis was significantly reduced in the *LoxC*-AS lines. The coincident reduction of both C5 and C6 volatiles is consistent with an essential role for a 13-lipoxygenase in synthesis of both sets of these fatty acid-derived volatiles. As a control for the emission of volatiles derived from independent biosynthetic pathways, the maintenance of wound-inducible (*E*)-caryophyllene emission demonstrates that *LoxC*-AS lines have no influence on terpenes (Fig. 5). Together, the results indicate a role for 13-LOX enzyme(s) in leaf GLV volatile synthesis. The large increase in *TomloxD* RNA in wounded tissues with reduced GLV emissions indicates that this 13-LOX is not involved in their synthesis. It is possible that it is involved in JA synthesis.

**Fig. 7.**
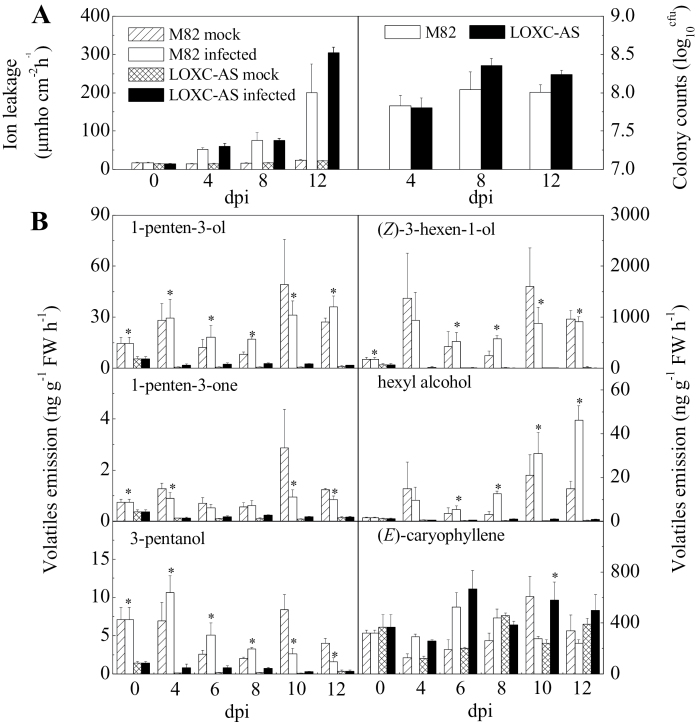
Leaf responses following *Xcv* infection. Plants were either infected with *Xcv* or mock-inoculated and sampled until complete leaf necrosis. (A) Ion leakage and bacterial colonization of infected leaves. (B) Release of selected C5 and C6 volatiles in M82 and *LoxC*-AS line plants challenged with *Xcv*. Volatiles were quantified on the indicated days after *Xcv* inoculation. (*E*)-caryophyllene is also shown as an example of an unrelated terpenoid volatile. Quantification of additional C5 and C6 volatiles is provided in Supplementary Fig. S2. Values are mean ± standard error of three replicates. Significant differences between M82-infected and *LoxC-AS*-infected plants are indicated with asterisks above bars: *P ≤ 0.05.

**Fig. 8.**
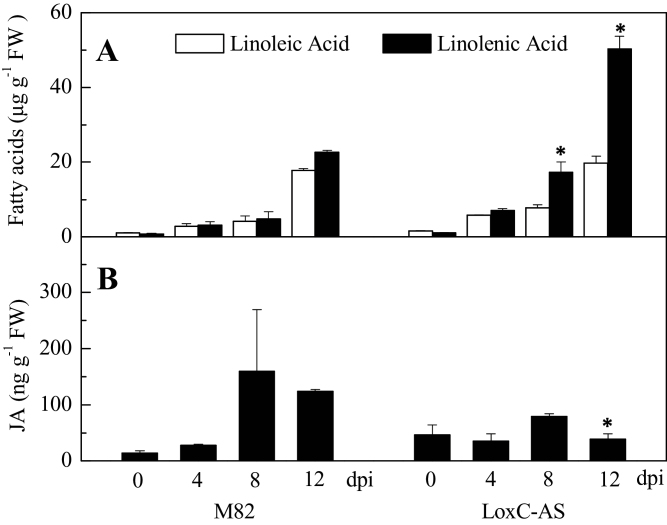
Quantification of jasmonic, linoleic, and linolenic acids following *Xcv* infection. Values are mean ± standard error of three biological replicates. Significant differences between M82-infected and *LoxC-AS*-infected plants are indicated with asterisks above bars: *P ≤ 0.05. Significant differences between *LoxC*-AS and M82 line plants at the same time points are indicated with asterisks above the bars: *P ≤ 0.05.

*LoxC*-AS line 0966 and its wild-type control (M82) were challenged with *Xcv* and the plants were maintained for 12 d after inoculation. During that period, the progression of disease was monitored by ion leakage and bacterial colony counts (Fig. 7A). Cell death, estimated by ion leakage, progressed up to 12 d after inoculation, at which point the leaves were fully necrotic. On average, *Xcv* growth in *LoxC*-AS 0966 was elevated compared to M82; however, the trend was not statistically significant (Fig. 7A). The emissions of GLVs by leaves of infected plants were monitored over the course of disease symptom development. As was observed in fruits, C5 and C6 leaf volatile emissions were also lower in the *LoxC*-AS lines (Fig. 7B, Supplementary Fig. S2). However, their synthesis was variable and did not significantly increase in response to *Xcv* infection. The very large reductions in GLV synthesis during symptom development did result in significantly higher accumulation of the fatty acid precursor linolenic acid (Fig. 8A).

This study group previously showed that JA is essential for *Xcv*-induced disease symptom development (O’Donnell *et al.*, 2003). Consistent with these prior results, there was increased JA accumulation following infection of the M82 plants (Fig. 8B). While there was detectable JA in the *LoxC*-AS leaves at all stages of the infection, there was no increase following *Xcv* infection. The data suggest that there is sufficient 13-LOX activity present to synthesize enough JA to permit symptom development. Antisense technology rarely achieves 100% knockout of the targeted gene. Whether the low level of JA detected is synthesized by residual TomloxC activity or that of the untargeted 13-LOX enzymes, TomloxD or TomloxF, is unknown.

It is concluded that *LoxC*-AS plants developed normal disease symptoms while exhibiting greatly reduced C6 and C5 volatile synthesis and somewhat reduced JA accumulation. Thus it is unlikely that these GLVs provide significant protection against *Xcv* infection.

## Discussion

Flavour quality in the fresh tomato is determined by the content of sugars, acids, and a diverse set of volatile chemicals derived from amino acids, fatty acids, and carotenoids (Klee, 2010). The complexity and multiplicity of independent metabolic pathways contributing to synthesis of flavour-associated chemicals makes quality improvement a significant challenge. In an effort to improve flavour, this study group has systematically sought to identify which of the many fruit chemicals are the most important determinants of consumer preferences (Tieman *et al.*, 2012). After identifying the most important chemicals, the pathways for synthesis, the genes encoding biosynthetic and regulatory activities, and alleles of the important genes that are capable of altering levels of the target compounds can be identified (Klee and Tieman, 2013). This work determined that C5 volatiles, including 1-penten-3-one, (*E*)-2-pentenal, 3-pentanone, 1-pentanol, and 1-penten-3-ol, are among the most highly associated with liking. Thus, identification of the pathway for their synthesis in the tomato fruit is essential for flavour improvement.

Previous work in other plant species has indicated that LOX activity is essential for generation of C5 volatiles (Salch *et al.*, 1995). A pathway involving two separate LOX reactions that generate first a hydroperoxide and then an alkoxyl radical has been proposed. The alkoxyl radical would undergo non-enzymic cleavage to generate C5 alcohols (Fig. 1). These alcohols can then be reduced to their corresponding aldehydes by alcohol dehydrogenases. If this proposed pathway is correct for tomato, then transgenic knockdowns of the essential LOX enzyme should be deficient in C5 volatiles while transgenic knockdowns of HPL should be either unaffected or potentially higher due to an increase in substrate pool size. This work observed both of these results. The 13-LOX *TomloxC* was targeted because it had previously been shown to be essential for synthesis of the C6 volatiles in tomato fruits (Chen *et al.*, 2004). There is only one *HPL* sequence in the tomato genome (http://solgenomics.net/). The data support the model; the *TomloxC* antisense lines had significant reductions in fruit and leaf C5 and C6 volatiles while *HPL*-reduced lines had significantly higher C5 volatile levels and significantly lower C6 volatiles in fruits. Since the antisense construct reduced *TomloxC* but not *TomloxD* or *TomloxF* transcripts in the fruit, it is concluded that synthesis of C5 flavour volatiles in ripe fruits is dependent upon TomloxC activity. Reductions in HPL activity increase the pool of the hydroperoxide, pushing 13-HPOT towards the C5 branch pathway (Fig. 1). It is important to note that the pathway cannot be entirely confirmed with the *LoxC*-AS lines since the enzyme is proposed to catalyse two separate reactions; without formation of 13-HPOT, the subsequent reaction would not occur.

The results presented here indicate that C5 volatiles are a component of the GLVs synthesized by leaves in response to wounding (Fig. 5). In contrast to the fruit results, this work cannot exclude a role for TomloxF activity since there were also significant reductions in both *TomloxF* and *TomloxC* RNAs. However, a role for *TomloxD* an be excluded, since its RNA was not altered by the antisense construct. Within the open reading frames, *TomloxF* is 78% identical to *TomloxC* while *TomloxD* is only 31% identical. In the context of pathogen challenge, it was somewhat surprising that there was no consistent pattern of induction of either C5 or C6 volatiles following *Xcv* infection (Fig. 7). GLVs are known to be induced during multiple plant–pathogen interactions (Croft *et al.*, 1993; Shiojiri *et al.*, 2006) and have been reported to have antimicrobial activity and/or induce defence responses (Bate and Rothstein, 1998; Matsui *et al.*, 2006; Scala *et al.*, 2013). While the current results are limited to the tomato-*Xcv* interaction, there was no induction of C5 and C6 volatiles following infection. The levels of both sets of volatiles were greatly reduced in leaves from the *LoxC-*AS plants. However this large reduction in the capacity for GLV production did not affect disease symptom formation.

The observed reductions in leaf-produced C5 and C6 volatiles are consistent with TomloxC and possibly TomloxF being the main 13-LOX enzyme(s) responsible for their synthesis. In contrast, there was significant, though reduced synthesis of JA following *Xcv* infection in the *LoxC*-AS plants. These results suggest that while TomloxC/F may contribute to JA synthesis, TomloxD is the major enzyme responsible for JA synthesis. In support of this hypothesis, four separate *Arabidopsis 13-Lox* gene products have been demonstrated to contribute to JA biosynthesis (Chauvin *et al.*, 2013). Similarly, in maize (*Zea mays*) ZmLOX10 is the primary biosynthetic enzyme responsible for GLV production; however, *ZmLOX10* mutants also display impaired JA production following wounding (Christensen *et al.*, 2013).

The discovery that loss of TomloxC function affects both C5 and C6 volatiles has caused this work to reassess some of the prior consumer evaluations of the antisense line (Tieman *et al.*, 2012). Multiple regression analysis of many heirloom tomato varieties led to the conclusion that C5 volatiles, but not C6 volatiles, are highly correlated with consumer liking. However, when consumers were specifically asked whether they preferred the transgenic line or the M82 control, there was no significant difference between the two varieties. This seeming contradiction is likely due to two causes. First, reductions in the C6 volatiles were relatively greater than those of the C5 volatiles. Second, M82, the background for the antisense experiments, is a processing variety and has relatively low levels of most flavour volatiles as compared to non-processing types of tomatoes. This group is now crossing the *TomloxC* antisense construct into heirloom varieties with much better flavour to reassess its effects on consumer preferences.

In conclusion, TomloxC has an essential function in synthesis of the important C5 flavour volatiles in both fruits and leaves. This synthesis is not dependent on HPL. The essential role of TomloxC in synthesis of volatiles that are highly correlated with consumer preferences indicates that screening for desirable *TomloxC* alleles for flavour improvement is justified. Synthesis of C5 volatiles increases in leaves following mechanical wounding but does not increase in response to *Xcv* infection. Large reductions in C5 and C6 volatiles in *LoxC*-AS plants were observed but those reductions did not alter the development of disease symptoms, indicating that these GLVs do not have an important defensive function against this bacterial pathogen.

## Supplementary material

Supplementary data are available at *JXB* online.

Supplementary Fig. S1. Phylogenetic analysis of lipoxygenases.

Supplementary Fig. S2. Release of C5 and C6 volatiles in M82 and *LoxC*-AS plants challenged with *Xcv*.

Supplementary Data
