# Supplementary material for: A 13-lipoxygenase, TomloxC, is essential for synthesis of C5 flavour volatiles in tomato
Source: J Exp Bot. 2014 Jan 22;65(2):419–28. doi: 10.1093/jxb/ert382 (PMC3904703; doi:10.1093/jxb/ert382)
Supplement: Supplementary Data [file supp_65_2_419__index.html]

A 13-lipoxygenase, TomloxC, is essential for synthesis of C5 flavour volatiles in tomato — A 13-lipoxygenase, TomloxC, is essential for synthesis of C5 flavour volatiles in tomato — Supplementary Data 

# A 13-lipoxygenase, TomloxC, is essential for synthesis of C5 flavour volatiles in tomato

## Supplementary Data

Data files

**Files in this Data Supplement:**

- Supplementary Data - Supplementary Data
