# Supplementary material for: A 13-lipoxygenase, TomloxC, is essential for synthesis of C5 flavour volatiles in tomato
Source: J Exp Bot. 2014 Jan 22;65(2):419–28. doi: 10.1093/jxb/ert382 (PMC3904703; doi:10.1093/jxb/ert382)
Supplement: Supplementary Data [file supp_ert382_jexbot106054_file001.pdf]

Supplementary materials

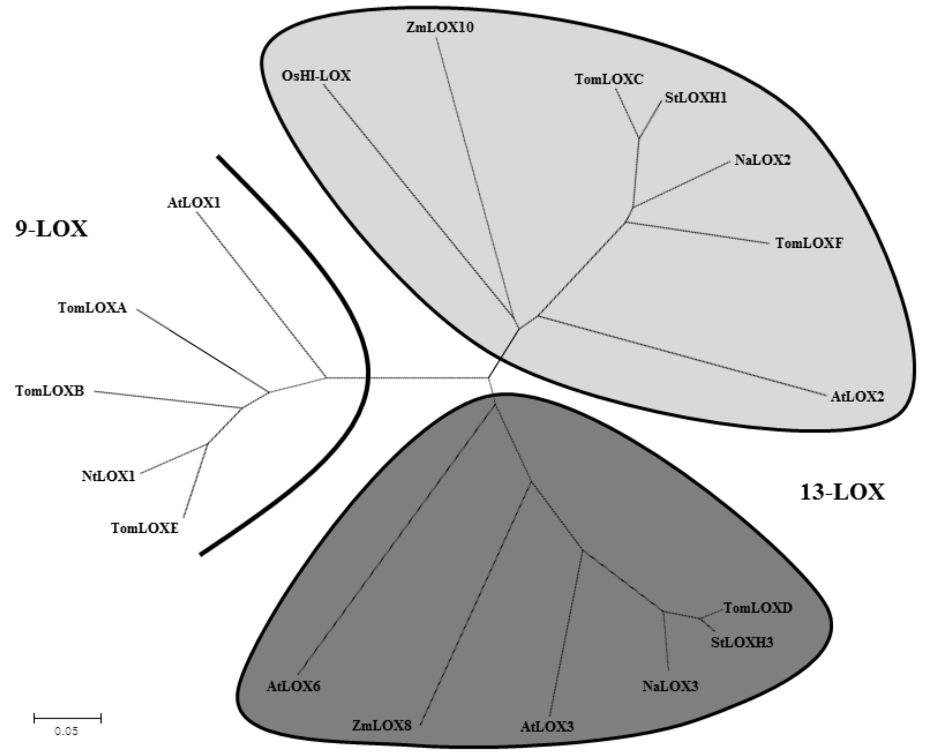

Supplementary Figure 1. Phylogenetic analysis of lipoxygenases (LOXs). The relationship between predicted amino acid sequences of tomato LOX enzymes and other species was analyzed with ClustalW and visualized with MEGA 5.1. The shaded areas correspond to two broad groupings of 13-lipoxygenase enzymes while the 9-lipoxygenases are unshaded. Accession numbers for the LOX sequences are: *S. lycopersicum* TomloxA (GenBank: AAA53184), TomloxB (GenBank: AAA53183), TomloxC (GenBank: AAB65766), TomloxD (GenBank: AAB65767) TomloxE (GenBank: AAG21691), TomloxF (GenBank: FJ617476); *S. tuberosum* StLOXH1 (GenBank: CAA65268), StLOXH3 (GenBank: CAA65269), *Nicotiana attenuata* NaLOX2 (GenBank: AAP83137), NaLOX3 (GenBank: AAP83138); *N. tabacum* NtLOX1 (GenBank: CAA58859); *Arabidopsis thaliana* AtLOX1 (GenBank: NP_175900), AtLOX2 (GenBank: AAL32689), AtLOX3 (GenBank: CAB56692), AtLOX6 (GenBank: CAG38328); *Oryza sativa* OsHI-LOX (GenBank: FJ607153), *Zea mays* ZmLOX8 (ts1) (GenBank: DQ335766/ FJ360855), ZmLOX10 (GenBank: DQ335768).

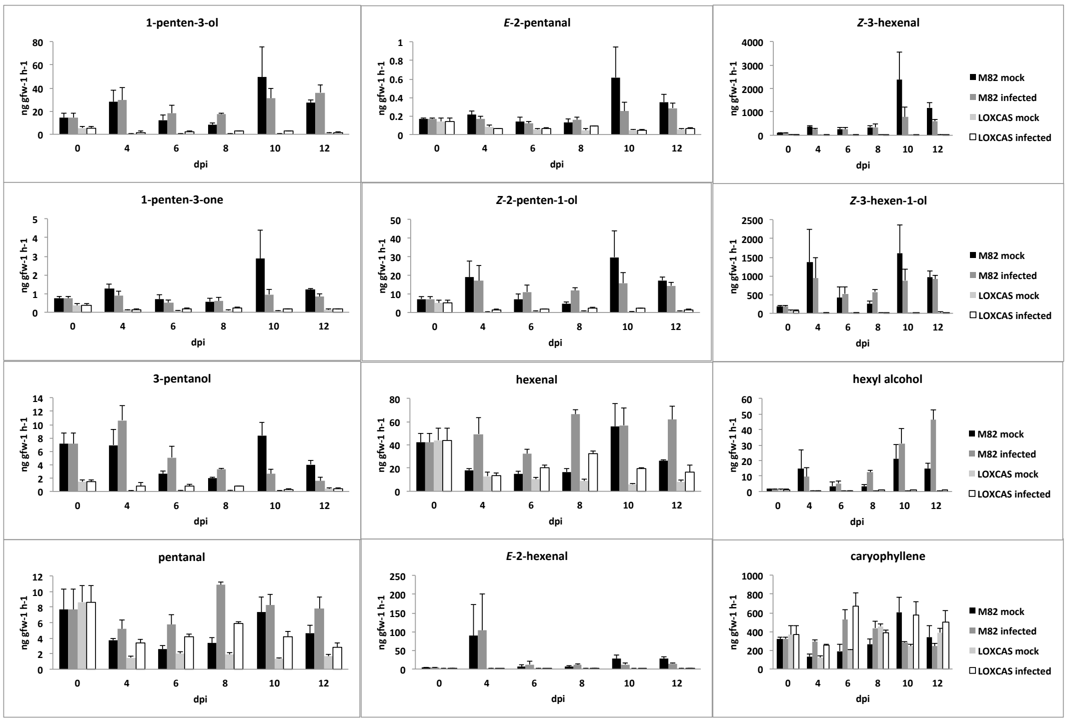

Supplementary Figure 2. Release of C5 and C6 volatiles in M82 and *LoxC*-AS line plants challenged with *Xcv*. Volatiles were quantified on the indicated days after *Xcv* inoculation. Caryophyllene is also shown as an example of an unrelated terpenoid volatile. Each point is the mean value of three replicates ± SE.
